# Enhanced YOLOv5 network-based object detection (BALFilter Reader) promotes PERFECT filter-enabled liquid biopsy of lung cancer from bronchoalveolar lavage fluid (BALF)

**DOI:** 10.1038/s41378-023-00580-6

**Published:** 2023-09-29

**Authors:** Zheng Liu, Jixin Zhang, Ningyu Wang, Yun’ai Feng, Fei Tang, Tingyu Li, Liping Lv, Haichao Li, Wei Wang, Yaoping Liu

**Affiliations:** 1 School of Software and Microelectronics, Peking University, Beijing, 100871 China; 2 Department of Pathology, Peking University First Hospital, Beijing, 100034 China; 3 School of Integrated Circuits, Peking University, Beijing, 100871 China; 4 Department of Respirology and Critical Care Medicine, Peking University First Hospital, Beijing, 100034 China; 5 Department of Interventional Lung Disease and Center of Endoscopic Diagnosis and Treatment, Anhui Chest Hospital, Hefei, Anhui 230022 China; 6 National Key Laboratory of Advanced Micro and Nano Manufacture Technology, Beijing, 100871 China; 7 Frontiers Science Center for Nano-optoelectronics, Peking University, Beijing, 100871 China; 8 AntiMicrobial Resistance (AMR) and Critical Analytics for Manufacturing Personalized-Medicine (CAMP) IRGs, Singapore-MIT Alliance for Research and Technology (SMART) Center, Singapore, 138602 Singapore

**Keywords:** Electrical and electronic engineering, Nanoscience and technology

## Abstract

Liquid biopsy of cancers, detecting tumor-related information from liquid samples, has attracted wide attentions as an emerging technology. Our previously reported large-area PERFECT (**P**recise-**E**fficient-**R**obust-**F**lexible-**E**asy-**C**ontrollable-**T**hin) filter has demonstrated competitive sensitivity in recovering rare tumor cells from clinical samples. However, it is time-consuming and easily biased to manually inspect rare target cells among numerous background cells distributed in a large area (*Φ* ≥ 13 mm). This puts forward an urgent demand for rapid and bias-free inspection. Hereby, this paper implemented deep learning-based object detection for the inspection of rare tumor cells from large-field images of PERFECT filters with hematoxylin-eosin (HE)-stained cells recovered from bronchoalveolar lavage fluid (BALF). CenterNet, EfficientDet, and YOLOv5 were trained and validated with 240 and 60 image blocks containing tumor and/or background cells, respectively. YOLOv5 was selected as the basic network given the highest mAP@0.5 of 92.1%, compared to those of CenterNet and EfficientDet at 85.2% and 91.6%, respectively. Then, tricks including CIoU loss, image flip, mosaic, HSV augmentation and TTA were applied to enhance the performance of the YOLOv5 network, improving mAP@0.5 to 96.2%. This enhanced YOLOv5 network-based object detection, named as BALFilter Reader, was tested and cross-validated on 24 clinical cases. The overall diagnosis performance (~2 min) with sensitivity@66.7% ± 16.7%, specificity@100.0% ± 0.0% and accuracy@75.0% ± 12.5% was superior to that from two experienced pathologists (10–30 min) with sensitivity@61.1%, specificity@16.7% and accuracy@50.0%, with the histopathological result as the gold standard. The AUC of the BALFilter Reader is 0.84 ± 0.08. Moreover, a customized Web was developed for a user-friendly interface and the promotion of wide applications. The current results revealed that the developed BALFilter Reader is a rapid, bias-free and easily accessible AI-enabled tool to promote the transplantation of the BALFilter technique. This work can easily expand to other cytopathological diagnoses and improve the application value of micro/nanotechnology-based liquid biopsy in the era of intelligent pathology.

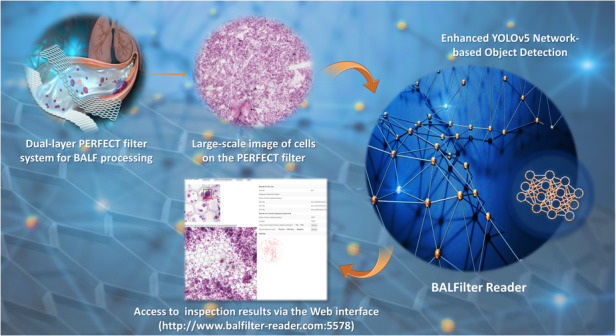

## Introduction

Lung cancer is the most lethal cancer worldwide^1^. Histopathology via biopsy is the current gold standard for the clinical diagnosis of lung cancer but suffers from disadvantages, including invasiveness, high risk, and limited accessibility. Recently, liquid biopsy (detection of tumor-related information, including cells, macrovesicles, nucleic acids and proteins from clinical liquids such as pleural effusion, blood, and bronchoalveolar lavage fluid (BALF)) has attracted wide attentions as an emerging technology. Detection of exfoliated tumor cells (ETCs) from BALF has been acknowledged as an effective approach to early diagnosis of lung cancer, with a history tracing back to as early as 1948 in the Journal of American Medical Association (JAMA)^2^. The traditional methods for recovering ETCs in clinics include centrifugation- and sedimentation-based methods, which suffer from low sensitivity^3^. During the past decades, given the rapid developments of micro/nanotechnology and interdisciplinary applications, the detection sensitivity of rare targets has been improved based on micro/nanotechnology and has shown good performance in lab demonstrations^3,4^. However, there is a gap from lab-ready techniques to hospital-applicable tools^5^. This gap is a major challenge in processing raw clinical samples, which are usually of a large volume and rare targets drowned in huge complex backgrounds. Therefore, high volume throughput capability is a key index for micro/nanotechniques to be well applied in clinics. Among them, the micropore-arrayed filtration has been acknowledged as the most promising to realize a high throughput in processing clinical samples^3^. Recently, a PERFECT (**P**recise-**E**fficient-**R**obust-**F**lexible-**E**asy-**C**ontrollable-**T**hin) filter technique was developed to sensitively detect rare exfoliated tumor cells from large-volume clinical BALF samples for lung cancer diagnosis, named as BALFilter. The BALFilter demonstrated a good performance in 33 clinical cases, with a much higher sensitivity (80.0%) than the routine cytocentrifuge (45.0%), based on the Hematoxylin-Eosin (HE) staining enabled morphological identification, with the histopathological results as the gold standard^5^. However, manually inspecting the rare ETCs on the PERFECT filter distributed in a large area (*Φ* ≥ 13 mm) suffers from some well-known deficiencies. First, the ratio of ETCs to background cells is quite low, which makes it difficult and time-consuming to identify rare tumor cells among numerous background cells. Second, the accuracy of manual inspection is easily biased resulting from uneven experience of pathologists. Artificial intelligence (AI) has been acknowledged as a promising tool to solve the abovementioned bottlenecks and has become a hot topic in the lab-on-a-chip community during the past decade^6–11^.

In recent years, deep learning technology has gradually matured and can quickly and accurately identify specific objects with the trained model from corresponding datasets^12–23^. Therefore, object detection based on deep learning has achieved wide applications in the field of tumor-related information identification for cancer diagnosis. Many researchers have used deep learning-based object detection on CT^24,25^, X-ray^16^, or other related images to realize the diagnosis of lung cancer^6,7,13,24^ or breast cancer^9,17,23^, with typical works summarized in Table 1.

**Table 1:** Recent typical works on deep learning-based object detection for cell recognition/cancer diagnosis

Ref. No.	Year	Network	Performance of network on MS COCO^a^	Modification(s) of the network	Trick(s)	Performance metric(s)	Source image	Applications
^12^	2022	SSD^b^	mAP^c^@0.5:0.95 (28.8%), mAP@0.5 (48.5%)	MobileNet used as the backbone	N/A	Accuracy (98%)	MRI images	Brain cancer
^13^	2020	RetinaNet	mAP@0.5:0.95 (40.8%), mAP@0.5 (61.1%)	N/A	N/A	AUC (0.87%)	X-ray images	Lung cancer
^14^	2022	YOLOv3	mAP@0.5:0.95(33.0%), mAP@0.5 (57.9%)	Dense block and S3Pool added in the backbone	Focal loss, K-means++, Soft NMS	mAP@0.5 (78.9%)	Microscope images (bright field)	Cervical cancer
^15^	2021	CenterNet	mAP0.5:0.95 (45.1%)	eSE module and bounding box attention unit added in backbone and head, respectively	N/A	mAP@0.5 (47.76%) on dataset A, mAP@0.5 (41.5%) on dataset B	CT images	Liver cancer
^16^	2021	EfficientDet-D7	mAP@0.5:0.95 (55.1%), mAP@0.5 (74.3%)	N/A	SFGAIA, HBBT, Rand Augment	Precision (42.5%) & Recall (90.2%) & F1 score (57.8%)	X-ray images	Gastric cancer
^17^	2022	YOLOv5	mAP0.5:0.95 (50.7%), mAP@0.5 (68.9%)	Parameters reduced in backbone	CLAHE	mAP@0.5 (96%)	X-ray images	Breast cancer
^18^	2021			N/A	N/A	mAP@0.5 (85.95%)	MRI images	Brain cancer
^19^	2021			SE block and concat block added in backbone and neck, respectively	N/A	mAP@0.5 (90%)	CT images	Lung cancer
^20^	2021			N/A	Mosaic, CIoU loss	mAP@0.5 (69.71%)	Microscope images (bright field)	Recognition of bone marrow cell
^21^	2021			N/A	N/A	mAP@0.5 (75%)	CT images	Lung cancer
^22^	2021			Assembling of YOLOv5-s and YOLOv5-m	CLAHE, rotation, random scaling, crop, flip, TTA	mAP@0.5 (62.7%)	Videolaryngo-scope images	Laryngeal cancer
^23^	2021	YOLOv4	mAP@0.5:0.95 (43.5%), mAP@0.5 (65.7%)	N/A	Mosaic, CIoU loss, label smoothing, DIoU loss	Sensitivity (88%)	Ultrasound images	Breast cancer
***This work***	***2022***	***YOLOv5***	***mAP0.5:0.95 (50.7%), mAP@0.5 (68.9%)***	N/A	***CioU loss, image flip, mosaic, HSV augmentation, TTA***	***mAP@0.5 (96.2%)***	***Microscope images (bright field)***	***Lung cancer***

The bold part highlights this work from the previously related reports

^a^MS COCO: a notable benchmark dataset

^b^SSD: single-shot detector

^c^mAP: mean average precision

Table 1 shows that CenterNet^26^, EfficientDet^27^ and YOLOv5^28^ are frequently used in cancer diagnosis and achieve higher performance on MS COCO (a notable benchmark dataset)^29^, which are higher than those of early classic networks such as single-shot detector (SSD)^30^, RetinaNet^31^, and YOLOv3^32^. Besides, various tricks have also been implemented to enhance the performance of basic networks, as shown in Table 1^14,16,17,20,22,23^. These tricks can be classified into two main types. The first type is mainly for data augmentation to improve the generalization of the basic network, which includes image flip, mosaic, mixup, HSV augmentation, rotation, random scaling, and crop. The other type is mainly developed to enhance the inference ability of basic networks with the frequently used tricks introduced as bellows. The contrast limited adaptive histogram equalization (CLAHE) is usually used in preprocessing to improve the local contrast and enhance the definitions of edges in each region of an image. K-means++ is mainly implemented to generate better anchor boxes by improving the performance in clustering. The intersection over union (IoU) loss function, including Generalized Intersection over Union (GIoU) Loss, Distance Intersection over Union (DIoU) loss, Complete Intersection over Union (CIoU) loss, can realize better bounding box regression (prediction). Focal loss is widely used to solve the imbalance in class (positive and negative samples) and classifications (easy and hard samples) via the dynamically scaled cross-entropy function (weighted cross-entropy loss) without modifying the dataset. Soft-non-maximum suppression (NMS) and weighted-NMS are applied for the deduplication of multiple bounding boxes generated in multiple rounds of inference. Test-Time Augmentation (TTA) can improve the accuracy by performing multiple rounds of inference.

In this work, YOLOv5, CenterNet and EfficientDet were first tested to select the optimal basic network. Subsequently, tricks including image flip, mosaic and HSV augmentation for data augmentation and CIoU/DIoU/GIoU loss, focal loss and TTA for inference enhancement were tested for performance improvement on the optimal basic network. Then, the optimal basic network and the performance-plus tricks constructed the BALFilter Reader, along with the customized Web (www.balfilter-reader.com:5578) for user-friendly interfaces. Finally, the established BALFilter Reader was tested on 24 clinical cases to validate its performance in the inspection of rare ETCs recovered on the PERFECT filters from BALF samples for the diagnosis of lung cancer.

## Experimental

**Fig. 1:**
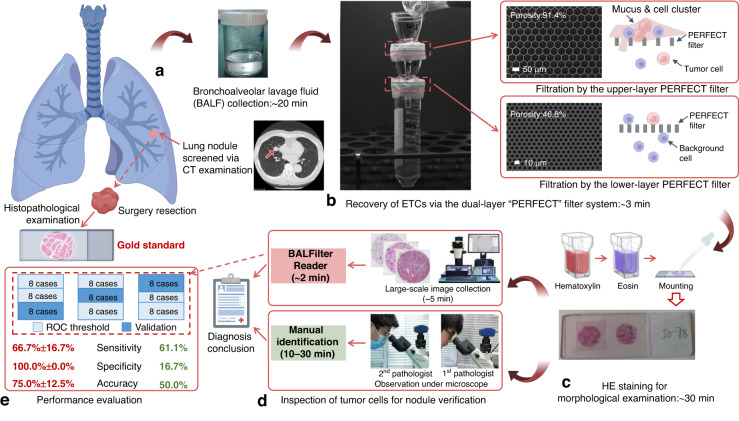
The overall workflow for the PERFECT filter-enabled liquid biopsy of lung cancer from BALF. **a** Collection of the BALF samples, **b**, **c** recovery of rare ETCs from BALF via the BALFilter technique, **d** identification of the recovered rare tumor cells via the AI-based detection (BALFilter Reader) and manual inspection as in-parallel comparisons, and **e** the evaluation of BALFilter Reader performance via cross-validation with 24 clinical cases

**Fig. 2:**
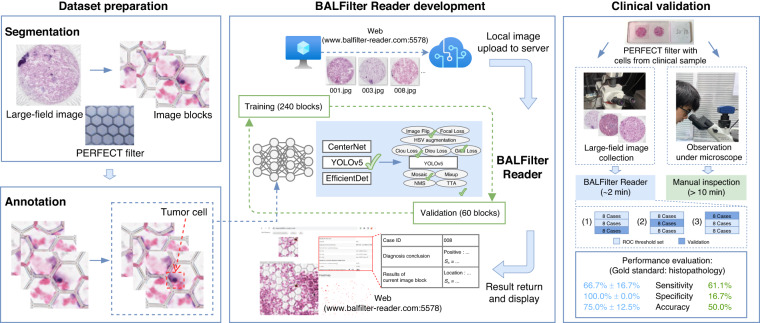
The schematic illustration of the workflow for the BALFilter Reader development. **a** Dataset preparation, **b** Annotation, **c** the development of BALFilter Reader, and **d** Clinical validation

Figure 1 schematically shows the overall workflow for the AI-based detection of rare tumor cells recovered by the PERFECT filter from BALF (**BALFilter Reader**) for the verification of lung nodule/diagnosis of lung cancer. The main procedures included three parts: (1) collection of the BALF samples (Fig. 1a), recovery of ETCs from BALF samples (Fig. 1b) and HE staining of the recovered ETCs in situ on the PERFECT filter (Fig. 1c); (2) detection of tumor cells from PERFECT filters via the BALFilter Reader based on the collected large-scale images with double-blind and duplicated manual inspections from two experienced pathologists as an in-parallel comparison, as shown in Fig. 1d; and (3) performance evaluation with the histopathological results as the gold standard (Fig. 1e), where 6 cases were negative and 18 cases were positive for lung cancer.

### Recovery of ETCs from clinical BALF samples and HE staining via the BALFilter technique

Clinical BALF samples were collected from lung cancer-suspected patients in Peking University First Hospital and Anhui Chest Hospital from June 2018 to February 2021. Patient recruitment was performed in compliance with the relevant laws and institutional guidelines (Good Clinical Practice (GCP) of China) under the IRB (2021034), approved by committees and signed by the chairman of the committee (Yanyan Yu) in Peking University First Hospital. The histopathological results were also collected from the department of pathology and taken as the gold standard for the diagnosis of lung cancer to evaluate the diagnostic performance of the BALFilter Reader and manual inspection. The processes for filtration of the BALF and Hematoxylin-Eosin (HE) staining of recovered cells followed our previously reported BALFilter technique^5^.

### Collection of large-field images of PERFECT filters with HE-stained cells recovered from clinical BALF samples

The large-field images (25,088*25,088 pixels) of PERFECT filters with HE-stained cells were collected under a microscope (DM6B^TM^, Leica) with the multi-position scanning function. Imaging was performed under a bright field over the whole effective filtration area (*φ*13 mm). As mentioned above, one large-field image of the BALFilter from a positive lung cancer BALF was used to establish the training and validation datasets for the BALFilter Reader. Large-field images from the other 24 clinical cases (6 negatives and 18 positives, with detailed information listed in Table S1 in the supplementary materials) were used to evaluate the performance of the developed BALFilter Reader.

### Development of the BALFilter Reader

The schematic illustration of the workflow for the BALFilter Reader development is shown in Fig. 2. First, the dataset was prepared with 300 image blocks from the segmentation of a typical large-field image of the PERFECT filter with HE-stained cells recovered from a positive clinical BALF sample. Among these image blocks, 80% (240) and 20% (60) were used for training and validation, respectively. In each image block, there were tumor cells and/or background cells annotated by two experienced pathologists. Second, the CenterNet, EfficientDet, and YOLOv5 networks were tested for optimal basic network decision according to the validation results. Subsequently, to further improve the performance of the basic network, various tricks, including image flip, mosaic and HSV augmentation for data augmentation and CIoU/DIoU/GIoU loss, focal loss and TTA for inference enhancement, were tested step by step, as listed in Table 2, with mAP@0.5 as the key metric for the effectiveness evaluation. The optimal basic network and performance-plus tricks constructed the BALFilter Reader. Moreover, a customized Web (browser App, www.balfilter-reader.com:5578) was developed as a friendly interface for users to easily upload the target images to the server where the BALFilter runs and view the returned detection results.

**Table 2:** Test of different tricks (combinations) for performance enhancement on the optimal basic network (YOLOv5-x)

Step	Objectives	Tricks (combinations)	mAP@0.5	Inclusion in the BALFilter Reader
1	Improving the intersection between the prediction and annotation bounding boxes	GIoU loss	92.1%	No
		DIoU loss	91.3%	No
		***CIoU loss***	***93.0%***	***Yes***
2	Data augmentation to improve the generalization of the basic network	CIoU loss & image flip	93.7%	No
		CIoU loss & image flip & mosaic	95.0%	No
		CIoU loss & image flip & mixup	92.0%	No
		***CIoU loss & image flip & mosaic & HSV augmentation***	***95.7%***	***Yes***
3	Improved recognition of poorly distinguishable objects	CIoU loss & image flip & mosaic & HSV augmentation & focal loss	94.7%	No
4	Deduplication of multiple bounding boxes	***CIoU loss & image flip & mosaic & HSV augmentation & default-NMS***	***95.7%***	***Yes***
		CIoU loss & image flip & mosaic & HSV augmentation & soft-NMS	95.7%	No
		CIoU loss & image flip & mosaic & HSV augmentation & weighted-NMS	95.7%	No
5	Overall performance improvement via multiple rounds of inference	***CIoU loss & image flip & mosaic & HSV augmentation & default-NMS & TTA (NMS)***	***96.2%***	***Yes***

The bold part is the combination of selected tricks for the best performance (highest mAP@0.5), which is also the focus of this work

### Segmentation

Given the large number of pixels of the large-field image, segmentation was necessarily performed to slice into ~10,000 image blocks (256*256 pixels for each) before starting the AI-enabled processing. For dataset preparation, 300 image blocks were selected from one typical positive clinical case. For the validation of the developed BALFilter Reader on 24 clinical cases, the sliced image blocks with obvious non-uniform staining, dye contamination, defocused cells or only micropores (no cells) were first filtered, and then the residual ones were used as the input for inference to identify the ETCs for the diagnosis of lung cancer.

### Annotation

The tumor cells and/or background cells in the 300 image blocks of the dataset were annotated by two experienced pathologists independently. If the two pathologists gave different conclusions on the same image in the independent inspections, discussion to get a joint conclusion was performed. Labellmg^33^ (version 1.8.1) and VGG Image Annotators (VIA, version 2.0.8)^34^ were used to generate annotations in the VOC format (labels are in xml format). Then, the VOC formatted dataset was converted into the YOLO, MS COCO, and TF Record formats via customized Python scripts, which were compatible with the mainstream networks for object detection. The annotated 300 image blocks were randomly divided into training (80%) and validation (20%) sets. In the training set, 89 image blocks were positive, containing tumor cells (and background cells), and 151 were negative, containing only background cells. In the validation set, there were 23 positives and 37 negatives.

### Enhancement of networks and tricks for BALFilter Reader construction

As shown in Fig. 2, the construction of the BALFilter Reader included the enhancement of the basic network and the selection of performance-plus tricks. First, the CenterNet, EfficientDet series, and YOLOv5 series networks^28,32,35,36^ with different model sizes^37^ were trained with the prepared training set to select the optimal basic network for the BALFilter Reader (detailed parameters are listed in Table S2 in the supplementary materials). Then, the trained networks were evaluated with the prepared validation set, with the mAP@0.5 as a key metric. All computing was conducted on a server with an AMD EPYC 7773 × 64-Core CPU, NVIDIA RTX A6000 GPU, and Torch version of 1.6.0+cu101. The selection of the optimal basic network was determined considering both the value of mAP@0.5 and the requirement for computing resources (i.e., the computational complexity).

**Fig. 3:**
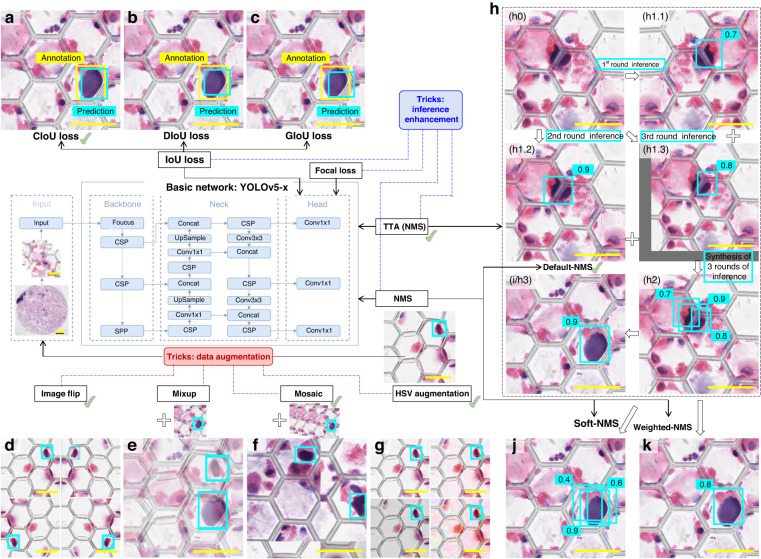
The tests of different techniques for performance enhancement on the optimal basic network (YOLOv5-x). The green-colored marked tricks are the selected ones for the construction of the BALFilter Reader. The scale bars in the image blocks and large-field images are 50 μm and 2 mm, respectively

Then, as shown in Fig. 3, to further improve the performance of the selected basic network, various tricks (listed in Table 2), including image flip, mosaic and HSV augmentation for data augmentation and CIoU/DIoU/GIoU loss, focal loss and TTA for inference enhancement, were tested, with the mAP@0.5 as a key metric to evaluate the effectiveness on the performance (increased or decreased value). Detailed description and values of hyper parameters for the tricks can be found in Table S3 of the supplementary materials.

Finally, the optimal basic network and performance-plus tricks constructed the BALFilter Reader. Moreover, a customized Web was developed to provide user-friendly interfaces. The operation of the BALFilter Reader via the developed Web (www.balfilter-reader.com:5578) is shown in video S1 of the Supplementary materials. All the source codes, installation packages and detailed instructions have been shared on GitHub (https://github.com/GROUPWW/balfilter-reader).

### Clinical validation

24 large-field images of PERFECT filters with HE-stained cells recovered from clinical BALF samples were input into the developed BALFilter Reader to perform tumor cell recognition for the diagnosis of lung cancer. Meanwhile, these PERFECT filters were also manually and independently inspected by two experienced pathologists to diagnose lung cancer. If the two pathologists gave different diagnosis conclusions for the same case in the independent inspections, discussion to get a joint conclusion was performed. The sensitivities, specificities and accuracies from the BALFilter Reader and manual inspection were calculated (Fig. 5a), taking the histopathological results as the gold standard.

**Fig. 4:**
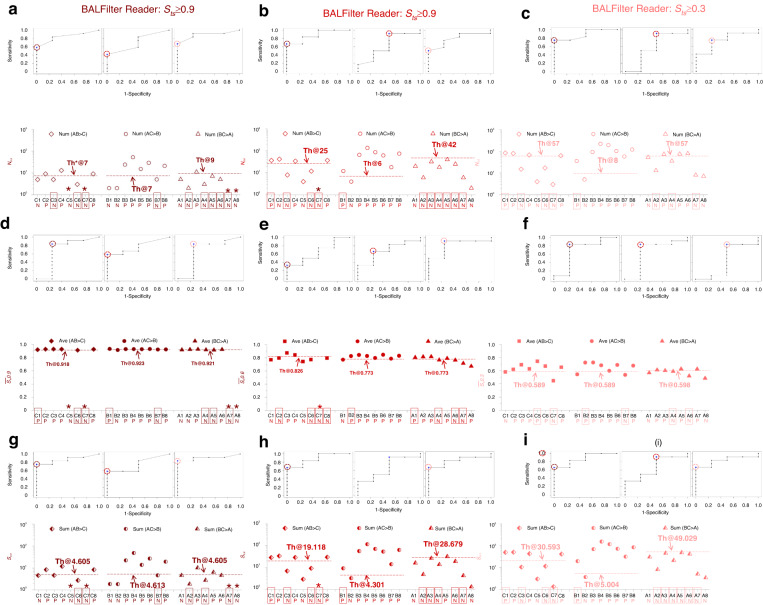
Tests of nine combinations (3 indicators × 3 classifications) to decide the optimal indicator and set the thresholds to distinguish the positive and negative cases for the diagnosis conclusion of clinical cases via cross-validation. The upper rows display the ROC curves from 2 groups (16 cases), and the lower rows show the diagnosis conclusions of the 3rd group (8 cases). The misdiagnosed cases by the BALFilter Reader are framed out with colored rectangles based on the indicators of the total number (num; *N*), the average value of *S*_*ts*_ (ave; $\bar{{{{S}}}_{{{ts}}}}$), and the sum (sum; *S* = $\bar{{{{S}}}_{{{ts}}}}$*N) for the three groups in (**a**–**c**), (**d**–**f**) and (**g**–**i**), respectively. *: there is no detected cell in the according classification

**Fig. 5:**
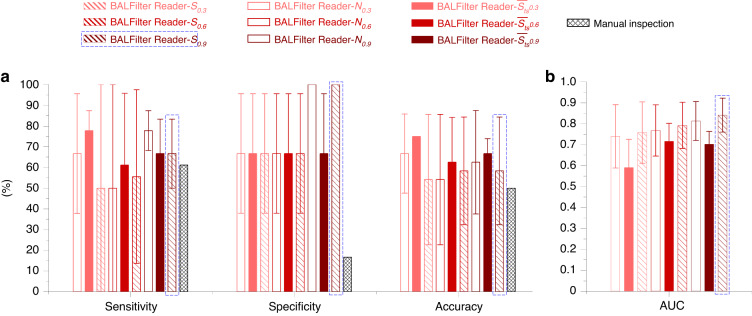
The evaluation of BALFilter Reader performance with clinical cases, with manual inspection as a parallel comparison. **a** Comparisons of the sensitivities, specificities, and accuracies obtained with the BALFilter Reader (based on nine different indicators) and the manual inspection. **b** The AUC values for the nine different indicators. The optimal indicator with the cutoff value of 0.9 (S_0.9_, based on the current results) was framed out with dashed blue rectangles

In the BALFilter Reader, the inference would generate the score(s) of tumor suspiciousness (*S*_*ts*_) for the predicted cells in each image block (displayed in the BALFilter Reader Web) and the heatmap with different colored dots reflecting the distribution of *S*_*ts*_ over the whole large-field image (Fig. S1 in the Supplementary materials). Then, different indicators for concluding the diagnosis results of the BALFilter Reader were investigated. First, the definition of tumor cells was tested with 3 different cutoff values (0.3, 0.6, 0.9) of *S*_*ts*_, i.e., 3 classifications (*S*_*ts*_ ≥ 0.3, *S*_*ts*_ ≥ 0.6 and *S*_*ts*_ ≥ 0.9). The total number of cells (num; *N*), the average value of *S*_*ts*_ (ave; $\bar{{{\boldsymbol{S}}}_{{\boldsymbol{ts}}}}$) and sum (sum; *S*, *i.e*., $\bar{{{\boldsymbol{S}}}_{{\boldsymbol{ts}}}}$*N) for each classification were extracted/calculated. The nine combinations of indicators were named *N*_*0.3*_, *N*_*0.6*_, *N*_*0.9*_, $\bar{{{\boldsymbol{S}}}_{{\boldsymbol{ts}}}}$_*0.3*_, $\bar{{{\boldsymbol{S}}}_{{\boldsymbol{ts}}}}$_*0.6*_, $\bar{{{\boldsymbol{S}}}_{{\boldsymbol{ts}}}}$_*0.9*_, *S*_*0.3*_, *S*_*0.6*_ and *S*_*0.9*_. Then, the 24 cases were divided into 3 groups (Groups A, B and C), with 8 cases (containing both histopathological positives and negatives) in each group, to cross-validate the performance of the BALFilter Reader and then estimate the errors of the sensitivities, specificities, and accuracies. 2 groups (16 cases) were used to produce the receiver operating characteristic (ROC) curves, as shown in Fig. 4, and define the thresholds (the colored circled points) for drawing the diagnosis conclusions in the 3rd group (8 cases), which are listed in Fig. 4. All incorrectly diagnosed cases are framed with colored rectangles. The sensitivity, specificity, and accuracy were calculated with the histopathological results as the gold standard of diagnosis. There were three combinations (AB>C, AC>B and BC>A) in the cross-validation, and all the calculated values were used to obtain the average and standard deviation for each indicator, as displayed in Fig. 5a, with those from the manual inspection as an in-parallel comparison. Moreover, the AUC (Area Under Curve) values for the above nine combinations were calculated from the ROC curves and displayed in Fig. 5b.

## Results and discussion

### Development of the BALFilter Reader

The first step in the development of the BALFilter Reader is the set of training/validation datasets. Each dataset should contain both positives and negatives. The ratios of positives vs. negatives in the training and validation sets were finally set after two runs of data collection. The first set had similar ratios of positives vs. negatives, where 89 positives and 91 negatives formed the training dataset, while 27 positives and 33 negatives formed the validation dataset. The following evaluation of the clinical samples showed poor performances on the negative cases. Considering that the recognition accuracy of negative cases is very important for cancer diagnosis to avoid false positive results causing anxiety to patients in clinical practices, another 60 negatives were added into the training dataset to further improve the performance of the BALFilter Reader on the negative cases. To further verify the performance and fulfill real clinical applications, the expansion of the dataset is a critical step. In dataset expansion, the balance between the ratios in the training and validation sets for positives vs. negatives and the percentage of negatives will be carefully considered and designed to improve the generation ability of the BALFilter Reader in clinical applications.

On the prepared training/validation dataset, the three networks were tested with different parameters (Table S2 in the supplementary materials). CenterNet achieved a mAP@0.5 of 85.2%. EfficientDet was tested with eight model sizes, and the achieved highest mAP@0.5 was 91.6%. YOLOv5 was tested with four different model sizes, and the achieved highest mAP@0.5 was 92.1%. After comparing the values of mAP@0.5 from the CenterNet, EfficientDet series and YOLOv5 series with different model sizes and considering the requirement for computing resources, YOLOv5-x was selected as the optimal basic network given its highest mAP@0.5 (92.1%) at an economy-friendly computing configuration.

As shown in Fig. 3, to further enhance the inference ability and improve the performance of the YOLOv5-x basic network, different tricks (combinations) were tested, with the values of mAP@0.5 collected on the validation set as the metric to decide inclusion or exclusion in the construction of the BALFilter Reader, as shown in Table 2. The selection of tricks was considered from the following five aspects. First, three classical regression functions, including GIoU, DIoU and CIoU (Fig. 3a–c), were tested to improve the degree of intersection between the prediction and annotation bounding boxes (i.e., making the prediction bounding box match the annotated tumor cells as well as possible). The CIoU loss was selected given its highest mAP@0.5 (93.0%) compared to those for GIoU loss and DIoU loss at 92.1% and 91.3%, respectively. Second, considering that the current dataset has a limited size, tricks including image flip (Fig. 3d), mixup (Fig. 3e), mosaic (Fig. 3f) and HSV augmentation (Fig. 3g) were tested for data augmentation while not affecting the pristine information in the image block to improve generalization of the basic network, with CIoU loss as the base. The combination of CIoU loss, image flip, mosaic, and HSV augmentation indicated the best performance with the most significantly increased mAP@0.5 at 95.7%. These data augmentation tricks contribute to improving the basic network’s ability to detect targeted cells with various staining statuses and morphological characteristics. Third, given the non-uniformity in HE staining, varieties of clinical samples and varied manual processes, there were some poorly-distinguishable cells with hard-to-recognize/classify characteristics in the large-field images of PERFECT filters. Therefore, the focal loss was expected to address the classification imbalance (hard and easy samples) problem by up-weighting the hard samples, i.e., focusing more on training a subset of hard examples, i.e., the abovementioned poorly distinguishable cells. However, the focal loss caused a decrement of mAP@0.5 (from 95.7% to 94.7%) based on the current dataset and was thereby excluded in the construction of the BALFilter Reader. Fourth, to deduplicate the multiple bounding boxes from different rounds of inference and finally generate/output one bounding box for each prediction, tricks including soft-NMS and weighted-NMS were tested to compare with the default-NMS imbedded in the YOLOv5-x network (Fig. 3i–k). The test results showed that Soft NMS and weighted-NMS achieved comparable effectiveness (same mAP@0.5 @95.7%) but did not result in the expected increment compared to default-NMS (mAP@0.5 @95.7%). Therefore, the BALFilter Reader used the default-NMS from the YOLOv5-x network for the deduplication of multiple bounding boxes. Finally, TTA (with default-NMS) was introduced to improve the overall inference ability via multiple rounds of inference (Fig. 3h) and improved the mAP@0.5 to 96.2%.

Above all, the YOLOv5-x network, enhanced by performance-plus tricks including CIoU loss, image flip, mosaic, HSV augmentation, default-NMS, and TTA (NMS), was set as the model of the deep learning-based object detection for inspection of rare tumor cells on the PERFECT filters recovered from BALF and stained by HE, i.e., the construction of the BALFilter Reader. The inspection time for a single image block is 20.5 ± 5.3 ms (calculated from 1500 image blocks). The time of the BALFilter Reader execution for one clinical case, including slicing, filtering, and inference, was 111.3 ± 26.6 s (calculated from 24 clinical cases). The fast running also indicated the high performance of the established model for the BALFilter Reader.

### User-friendly interface of the BALFilter Reader

Moreover, the customized Web (www.balfilter-reader.com:5578) was also developed to provide user-friendly interfaces to guarantee an easy operation among users, including AI professionals, BALFilter technique developers and clinical doctors. The access to the BALFilter Reader through the customized Web is demonstrated in Video S1 in the supplementary materials. The collected large-field images could be easily uploaded to the server for BALFilter Reader analysis via the Web, followed by the initiation of detection (running the BALFilter Reader). The results from the BALFilter Reader interference are returned and can be easily viewed on the Web. The detection results mainly include two parts. One is the overall diagnosis conclusion of the case (positive or negative), and the other is detailed information, including the average value *S*_*ts*_ ($\bar{{S}_{{ts}}}$), the total number (*N*) and the sum (*S*, $\bar{{S}_{{ts}}}$*N)) of predicted cells for the three classifications. Besides, single image block can be easily addressed and viewed on the Web, which provides an easy access for pathologists to view the adjacent cells/microenvironments around the predicted cells and thus better check the correctness of inference of the BALFilter Reader. Then, the double-checked objects (predicted tumor cells or background cells by the BALFilter Reader) can be submitted/added into the original dataset via the link provided on the Web, which is a simple and efficient method to further expand the dataset with more clinical samples in future.

### Clinical validation

The BALFilter Reader was tested on 24 clinical cases (detailed information listed in Table S1 in the supplementary materials) to verify its application performance in the diagnosis of lung cancer based on our previously reported BALFilter technique^5^. The input of the BALFilter Reader is the large-filed image of the PERFECT filter with HE-stained cells recovered from clinical BALF samples, and the output includes 1) overall diagnosis (positive or negative) for the case, 2) detailed information about *S*_*ts*_ for predicted cells, and 3) a heatmap displaying the distribution of *S*_*ts*_ over the whole large-field image. Thereinto, the conclusion of the overall diagnosis for the case based on the inferred *S*_*ts*_ was carefully investigated, as mentioned above. 9 combinations of indicators, including *N*_*0.3*_, *N*_*0.6*_, *N*_*0.9*_, $\bar{{S}_{{ts}}}$_*0.3*_, $\bar{{S}_{{ts}}}$_*0.6*_, $\bar{{S}_{{ts}}}$_*0.9*_, *S*_*0.3*_, *S*_*0.6*_ and *S*_*0.9*,_ were all tested in the abovementioned cross-validation method to select the optimal index and set the threshold to distinguish positive and negative cases for the conclusion of diagnosis, as shown in Fig. 4a–i. The thresholds were chosen based on the overall consideration of both sensitivity and specificity. The calculated sensitivities, specificities, and accuracies for the above 9 combinations and manual inspection, with the histopathological results as the gold standard, are shown in Fig. 5a. Moreover, AUC values calculated from the ROC curves for the above 9 combinations are shown in Fig. 5b.

From the diagnosis results from 24 clinical cases shown in Fig. 4, the diagnosis performance varied among different indicators in different classifications. Combining the average values and standard deviations obtained from the cross-validation, the three indexes (sensitivity, specificity, accuracy) present no obvious trend for the indicators of *N* and $\bar{{S}_{{ts}}}$ but elevate for *S*, with the increment of the *S*_*ts*_ cutoff value among the three classifications. The AUC value for each indicator elevates with the increment of *S*_*ts*_ cutoff value. The preliminary results in Fig. 5 show that the combinative strategy with the sum from the group of *S*_*ts*_ ≥ 0.9, *S*_*0.9*_, is the optimal indicator to draw the diagnosis conclusion for the BALFilter Reader, given its best overall performance with sensitivity@66.7% ± 16.7%, specificity@100.0% ± 0.0% and accuracy@75.0% ± 12.5%, and highest AUC value (0.84 ± 0.08) obtained from ROC curves in the cross-validation. Therefore, the diagnosis conclusion based on the *S*_*0.9*_ was taken as the claimed performance of the BALFilter Reader to compare with that from the manual inspection in this paper, unless specified. The combinative strategy takes both the level of tumor suspiciousness of cells and the number of tumor/tumor-suspected cells into consideration, which is the actual process of inspection by a pathologist in clinical practices. With *S* as the optimal indicator and a cutoff value of 0.9, i.e., S_*0.9*,_ to distinguish positive and negative cases (Fig. 5a), the diagnostic performance of the BALFilter Reader is superior to that of manual inspection (sensitivity@61.1%, specificity@16.7% and accuracy@50.0%), which can be further verified by the following typical cases.

**Fig. 6:**
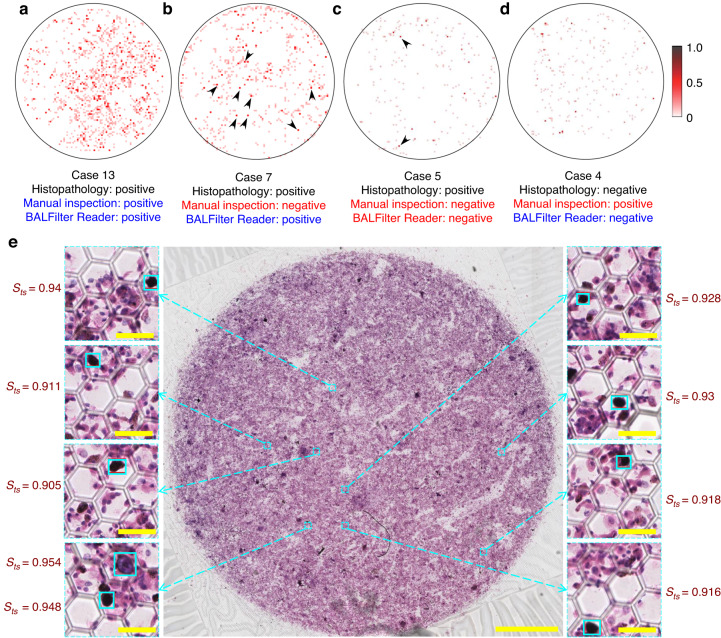
The typical clinical cases to demonstrate the advantage of the BALFilter Reader compared to the manual inspection. **a**–**d** Heatmaps of the typical cases (13, 7, 5, and 4, respectively) generated by the BALFilter Reader. The dot color deepens with the increment of *S*_*ts*_ value. The black arrows marked deep-red dots are those cells/positions with *S*_*ts*_ ≥ 0.9 predicted by the BALFilter Reader. **e** The large-field image of the PERFECT filter with HE-stained cells recovered from the BALF of histopathology-positive Case 7. The scale bar for the large-field image is 2 mm, and those for the inset image blocks are 50 μm

For the histopathology-positive Case 13 (with the highest value of *S*_*0.9*_), both the BALFilter Reader and manual inspection concluded the correct diagnosis (true-positive), which reveals that the diagnosis is relatively easy for the case with abundant ETCs in BALF (deep-red dots of *S*_*ts*_ ≥ 0.9 in the heatmap shown in Fig. 6a). Given the intrinsic advantage of AI in multi-tasking in a short time with reduced error, the development of AI-based techniques should focus more on pursuing an accurate diagnosis for challenging cases where there are only a small number and a low relative ratio (*i.e*., abundance) of ETCs among a large number of background cells, thereby bias easily appearing in the manual inspection, such as Cases 7 and 5 shown in Fig. 6b, e and c, respectively. For the histopathology-positive Case 7 with the heatmap and large-field image shown in Fig. 6b and e, respectively, the manual inspection concluded a false-negative diagnosis. Nevertheless, the BALFilter Reader reported a true-positive conclusion, although with only 9 cells of *S*_*ts*_ ≥ 0.9 (marked with black arrows) predicted, which were randomly scattered in 8 positions over the whole effective filtration area (8 out of more than 1500 image blocks). It is not hard to understand how difficult for pathologists to identify a small number of tumor cells, even with extensive observation under a microscope in a practically acceptable time in clinics. However, for the histopathology-positive Case 5, the cytopathology examination based on the BALFilter technique was concluded as negative by both the BALFilter Reader and manual inspection. From the heatmap shown in Fig. 6c, there were only 2 cells of *S*_*ts*_ ≥ 0.9 (marked with black arrows) predicted by the current BALFilter Reader, generating a false-negative conclusion based on the abovementioned index. The heatmap of Case 5 is difficult to virtually differentiate from that of histopathology-negative Case 4 (Fig. 6d). The obvious difference between Case 13 (histopathology positive) and Case 4 (histopathology negative), but the indistinguishable difference between Case 5 (histopathology positive) and Case 4, indicates that the incorrectness of diagnosis (false negative) from both the BALFilter Reader and manual inspection probably resulted from the very small number and the very low relative ratio of ETCs on the PERFECT filter. What is worth mentioning is that it is still promising to achieve further improvement in performance for the BALFilter Reader (upgraded version) given the rapid development and update in the field of AI, which is exactly the key point we will put efforts into with expanded dataset and optimized algorithms in the ongoing work. Moreover, another point that cannot be neglected is that the optimization of the key index and the setting of the threshold to better distinguish the positive and negative cases (i.e., draw more accurate diagnosis conclusions for the BALFilter Reader) in our future work needs careful investigations with more clinical cases.

Last not the least, the diagnosis based on the developed BALFilter Reader (~2 min) is much more rapid than the manual inspection from even an experienced pathologist (usually 10–30 min, occasionally even longer than 30 min for some poorly distinguishable cases). The short progressing time, along with the ease of operation, will promote the application expansion of the BALFilter Reader from regular cytopathological inspection to rapid intraoperative diagnosis and fulfillment of a high-performance diagnosis in resource-limited remote areas based on the efficient BALFilter technique.

Above all, the BALFilter Reader is believed to be of great use to fulfill rapid and bias-free diagnosis after careful training with the constantly optimizing model on the ever-expanding and ever-updating dataset. The carefully optimized and upgraded BALFilter Reader will promote the transplantation of the BALFilter technique for wide applications in clinical practices and thus improve the application value of emerging micro/nanotechnology based liquid biopsy.

## Conclusions

This work developed an artificial intelligence (AI)-assisted automatic, rapid, and bias-free recognition of rare tumor cells from large-field images of cells on the PERFECT filter (BALFilter Reader) to promote the wide application of the BALFilter-based liquid biopsy of lung cancer. Various tricks, including image enhancement methods such as image flip, mosaic, HSV augmentation and mixup, were implemented to improve cell recognition performance of the YOLOv5 network. Meanwhile, by modifying the original code, the YOLOv5 performances with different IoU methods, including DIoU/GioU/CioU, were also tested. In addition, some tricks to further improve the accuracy of cell recognition, including TTA and various NMS methods (weighted NMS and soft NMS), were investigated. By implementing the above tricks and adjusting the related parameters in the YOLOv5 network training process, as shown in Table 1, mAP@0.5 up to 96.2% was successfully achieved in recognition of rare tumor cells on the large-area PERFECT filter. The whole cell recognition process can be finished within 2 min. In the cross-validation with 24 clinical cases, the overall diagnosis performance with sensitivity@66.7% ± 16.7%, specificity@100.0% ± 0.0% and accuracy@75.0% ± 12.5% achieved by the BALFilter Reader is superior to that from the time-consuming (10–30 min) manual inspection with sensitivity@61.1%, specificity@16.7% and accuracy@50.0%, with histopathological results as the gold standard. The AUC value of the BALFilter Reader is 0.84 ± 0.08. Moreover, the customized Web makes the BALFilter Reader user-friendly and will promote wide usage. The Web interface can make the user easily upload the source image to the server where the BALFilter Reader runs and views the returned detection result. The achieved results showed that the developed BALFilter Reader, although just at the beta version, is a rapid, bias-free and easily accessible AI-enabled tool to promote the transplantation of the BALFilter technique. This work can easily expand to other cytopathological diagnoses and improve the application value of micro/nanotechnology based liquid biopsy in the era of intelligent pathology.

Expansion of the dataset is very important to further improve the performance of the BALFilter Reader. The customized Web will provide an easy way for pathologists to identify tumor cells/background cells, annotate image blocks and expand the dataset. The ratio of negatives vs. positives in the dataset needs careful design and optimization to improve the generation ability of the model. The optimization of key indicators and the setting of thresholds to better distinguish positive and negative cases and draw more accurate diagnostic conclusions also need careful investigations with more clinical cases. Moreover, the quality of the source image is also nonnegligible. Therefore, standardized sample preparation of clinical samples should be carefully designed to guarantee high-quality large-field images, especially in multicenter studies, to verify the universal applicability of the BALFilter technique in different levels of hospitals and clinical laboratory centers.

### Supplementary information

Supplementary material

video S1
