# Supplementary material for: Enhanced YOLOv5 network-based object detection (BALFilter Reader) promotes PERFECT filter-enabled liquid biopsy of lung cancer from bronchoalveolar lavage fluid (BALF)
Source: Microsyst Nanoeng. 2023 Sep 29;9:121. doi: 10.1038/s41378-023-00580-6 (PMC10541878; doi:10.1038/s41378-023-00580-6)
Supplement: Supplementary file 1 — Supplementary material [file 41378_2023_580_MOESM1_ESM.docx]

**Enhanced YOLOv5 Network-based Object Detection (BALFilter Reader) Promotes PERFECT Filter-Enabled Liquid Biopsy of Lung Cancer from Bronchoalveolar Lavage Fluid (BALF)**

Zheng Liu#, a, Jixin Zhang#, b, Ningyu Wang#, c, Yun’ai Fengd, Fei Tange, Tingyu Lic, Liping Lve, Haichao Li,*d Wei Wang*c, f, g and Yaoping Liu*c, h

# These authors contributed equally to this work.

**AUTHOR INFORMATION**

a. School of Software and Microelectronics, Peking University, Beijing 100871, China.

b. Department of Pathology, Peking University First Hospital, Beijing 100034, China.

c. School of Integrated Circuits, Peking University, Beijing 100871, China. E-mail: [w.wang@pku.edu.cn](mailto:w.wang@pku.edu.cn)

d. Department of Respirology and Critical Care Medicine, Peking University First Hospital, Beijing 100034, China. E-mail: [lhch91767@sina.com](mailto:lhch91767@sina.com)

e. Department of Interventional Lung Disease and Center of Endoscopic Diagnosis and Treatment, Anhui Chest Hospital, Anhui 230022, China

f. National Key Laboratory of Advanced Micro and Nano Manufacture Technology, Beijing 100871, China. E-mail: [w.wang@pku.edu.cn](mailto:w.wang@pku.edu.cn)

g. Frontiers Science Center for Nano-optoelectronics, Peking University, Beijing 100871, China. E-mail: [w.wang@pku.edu.cn](mailto:w.wang@pku.edu.cn)

h. AntiMicrobial Resistance (AMR) and Critical Analytics for Manufacturing Personalized-Medicine (CAMP) IRGs, Singapore-MIT Alliance for Research and Technology (SMART) Center, 138602, Singapore. E-mail: liu.yaoping@smart.mit.edu, [yaopingliu@pku.edu.cn](mailto:yaopingliu@pku.edu.cn)

# Fig. S1: The heatmaps of 24 clinical cases generated by the BALFilter Reader

The heat map which displays the distribution of *S_ts_* of the predicted cells over the whole effective filtration area of the PERFECT filter was generated by the BALFilter Reader after the running of inference. The dot color deeps with the increment of *S_ts_* value.

| 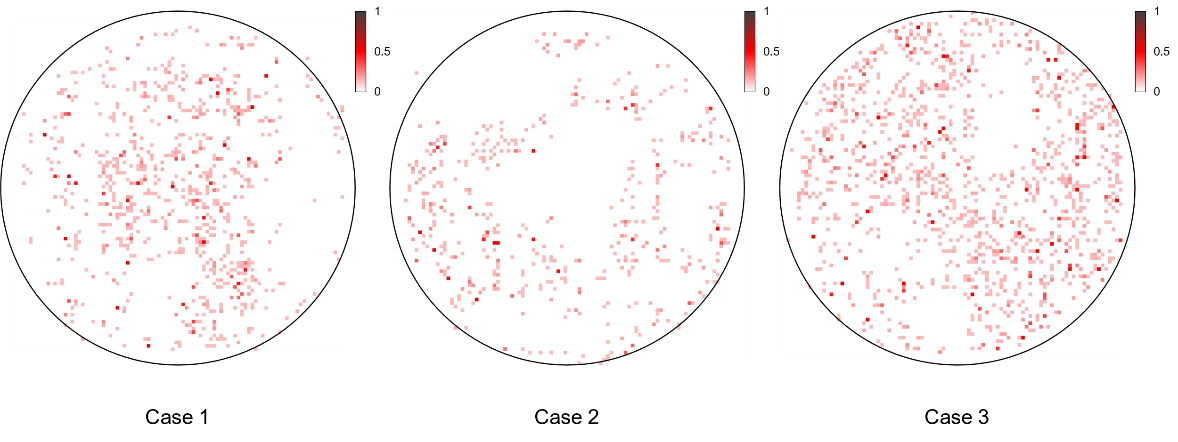 |
| --- |
| 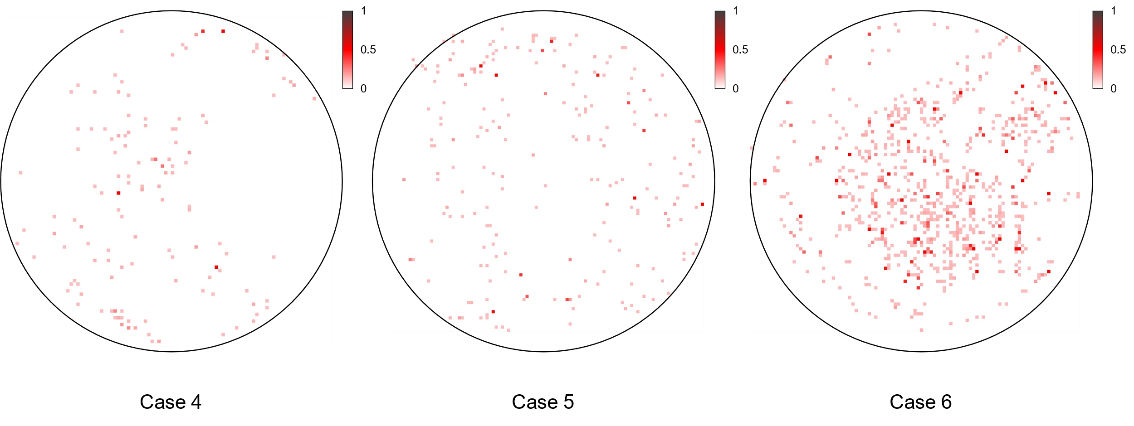 |
| 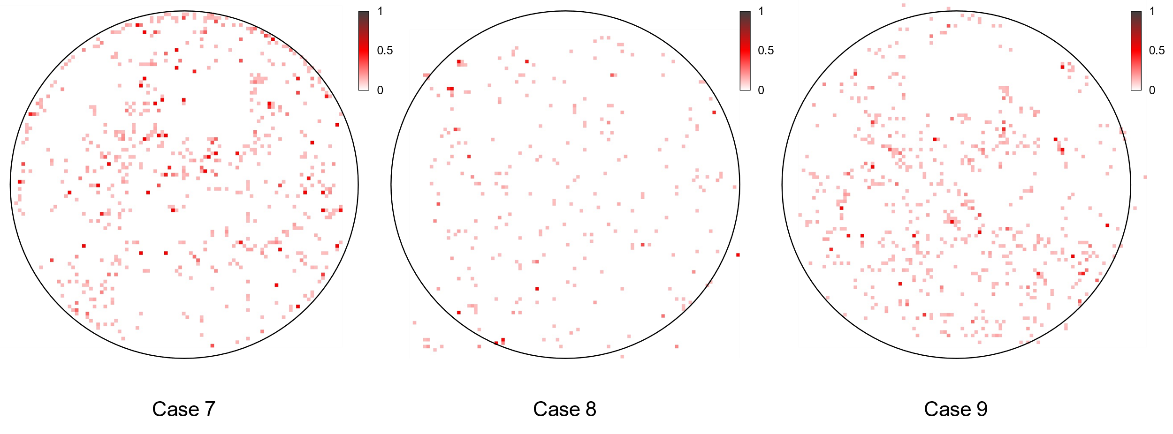 |
| 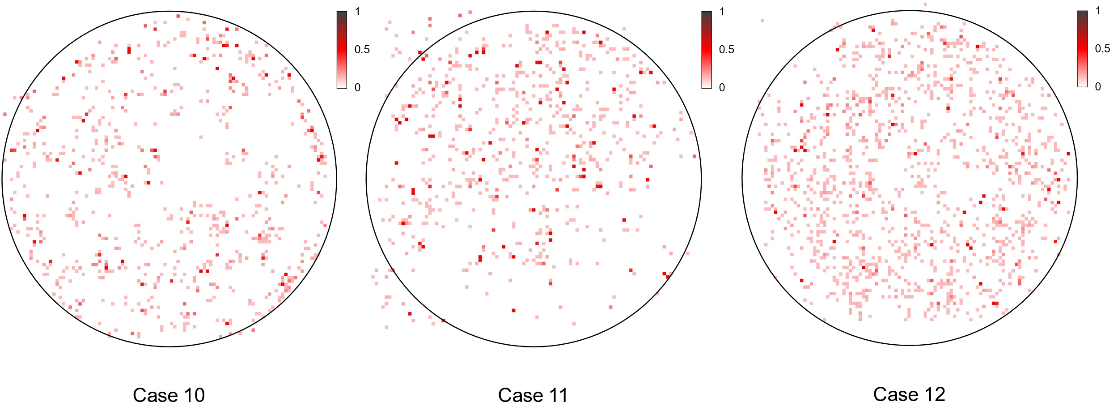 |
| 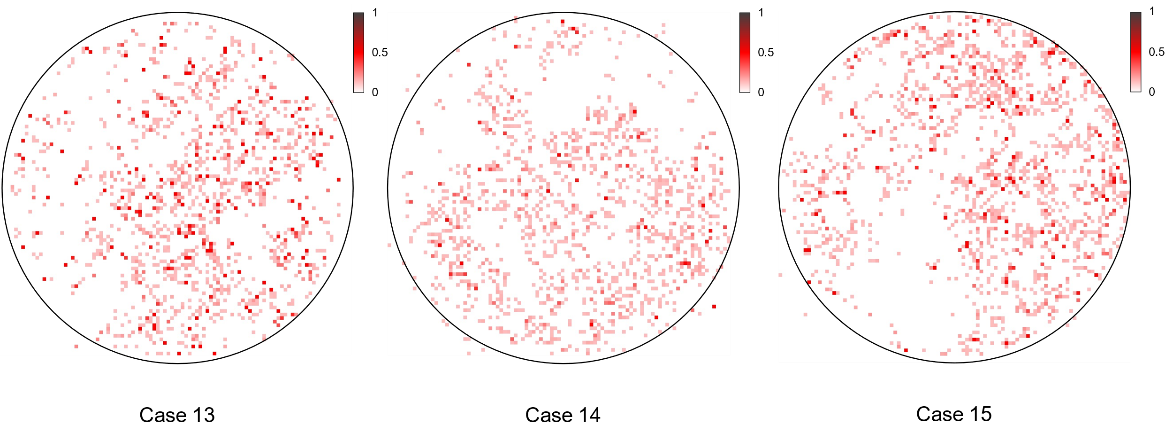 |
| 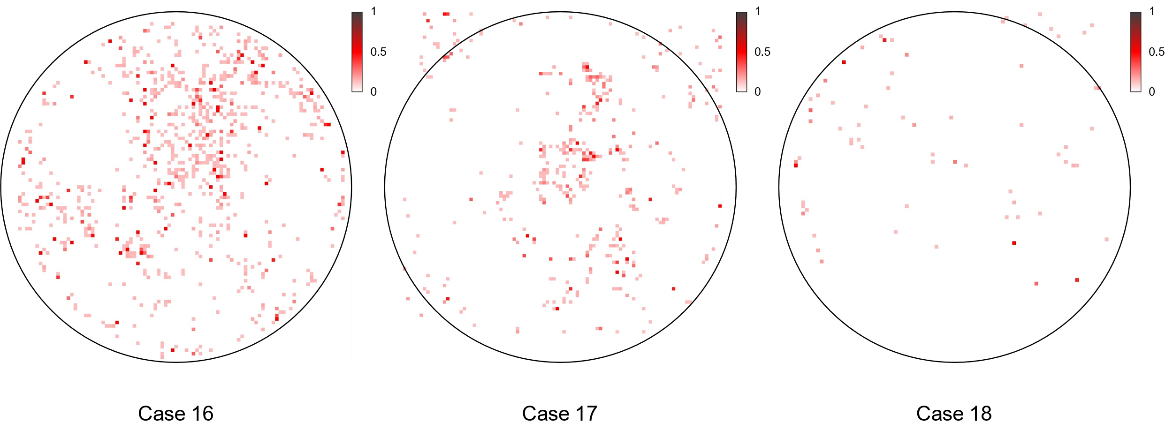  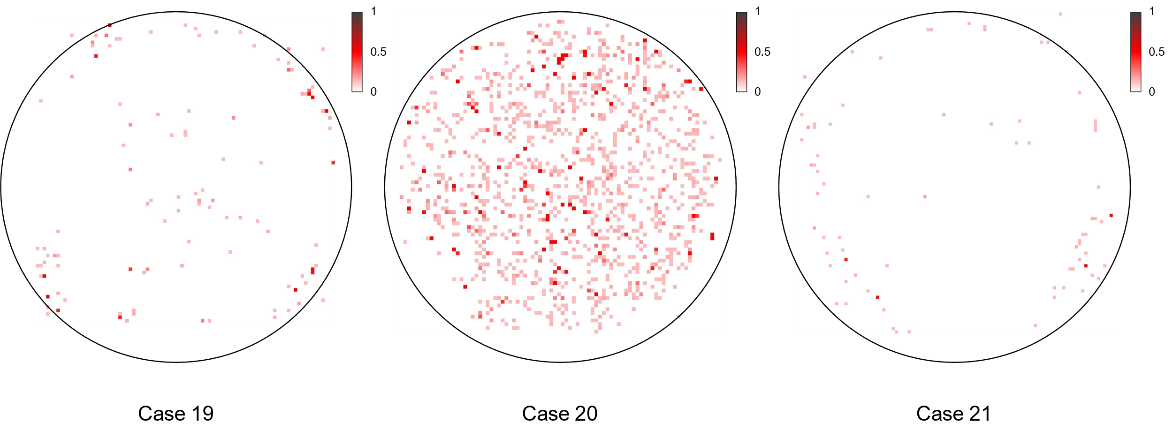  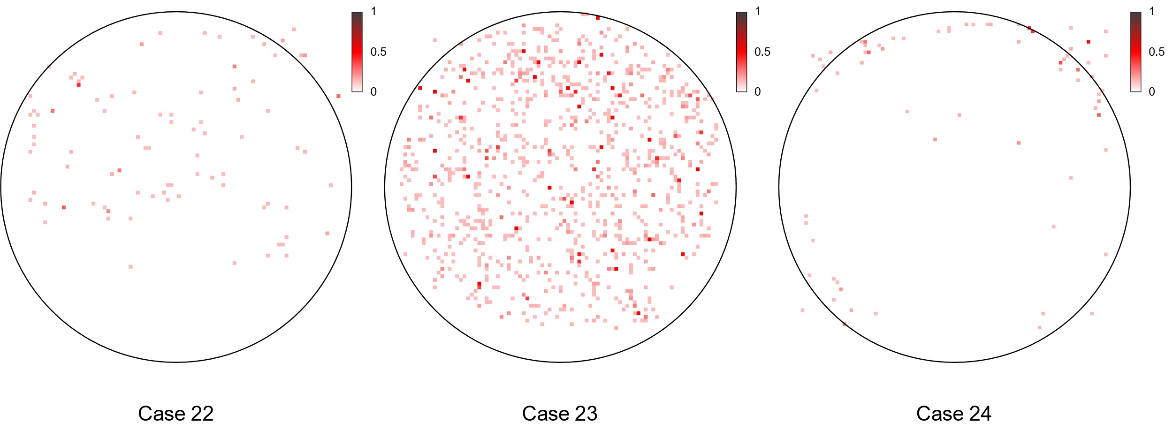 |

**Fig. S1** The heat maps of 24 clinical cases generated by the BALFilter Reader.

# Fig. S2: Typical false positive inference examples without adding additional negative images in the training set


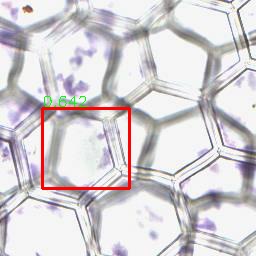


http://www.balfilter-reader.com:5578/result/001_without60/44


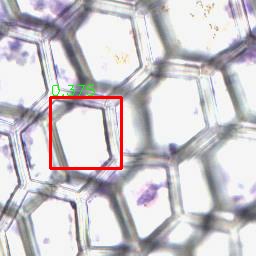


http://www.balfilter-reader.com:5578/result/001_without60/68

# Table S1: Detailed information of the clinical cases

The information for all the 25 clinical cases (1 for dataset preparation and 24 for clinical validation of the performance of the BALFilter Reader) involved in this work.

**Table S1** Detailed information of 25 clinical cases

| Usage. | Age | Gender | Histopathological  results | Manual inspection  results | BALFilter Reader  results | Cross-validation Group |
| --- | --- | --- | --- | --- | --- | --- |
| Dataset preparation | 64 | Female | Positive | - | - | - |
| Clinical verification | 53 | Female | Negative | Positive | Negative | A |
|  | 50 | Male | Negative | Positive | Negative | B |
|  | 45 | Female | Negative | Negative | Negative | C |
|  | 55 | Male | Negative | Positive | Negative | B |
|  | 73 | Female | Positive | Negative | Negative | A |
|  | 66 | Female | Positive | Negative | Positive | A |
|  | 54 | Male | Positive | Negative | Positive | C |
|  | 33 | Female | Positive | Negative | Negative | C |
|  | 67 | Male | Positive | Positive | Negative | A |
|  | 54 | Male | Positive | Positive | Positive | A |
|  | 57 | Male | Positive | Positive | Positive | B |
|  | 57 | Female | Positive | Positive | Positive | C |
|  | 76 | Female | Positive | Positive | Positive | B |
|  | 59 | Female | Positive | Positive | Negative | A |
|  | 56 | Female | Positive | Positive | Positive | B |
|  | 43 | Female | Positive | Positive | Positive | B |
|  | 30 | Male | Positive | Positive | Negative | B |
|  | 64 | Female | Positive | Positive | Negative | A |
|  | 40 | Male | Positive | Negative | Negative | C |
|  | 48 | Male | Positive | Negative | Positive | B |
|  | 69 | Female | Negative | Positive | Negative | C |
|  | 70 | Female | Positive | Negative | Negative | C |
|  | 41 | Male | Positive | Positive | Positive | C |
|  | 65 | Male | Negative | Positive | Negative | A |

# Table S2: Detailed parameters in the tested networks

**Table S2** The detailed parameters and test results of different networks

| Networks | Parameters | | | | | mAP  @0.5 | |
| --- | --- | --- | --- | --- | --- | --- | --- |
| CenterNet1 (r1) | The layer number in ResNet | | | The number of convolution kernels | | |  |
|  | 152 | | | 3, 8, 36, 3 | | | 85.20% |
| EfficientDet2 (r2) | Backbone | Layer number of BiFPN-C | Layer number of  BiFPN-L | | Layer number of Box/Class-L |  | |
|  | B0 | 64 | 3 | | 3 | 87.60% | |
|  | B1 | 88 | 4 | | 3 | 90.40% | |
|  | B2 | 112 | 5 | | 3 | 91.60% | |
|  | B3 | 160 | 6 | | 4 | 91.30% | |
|  | B4 | 224 | 7 | | 4 | 91.00% | |
|  | B5 | 288 | 7 | | 4 | 90.60% | |
|  | B6 | 384 | 8 | | 5 | 83.20% | |
|  | B7 | 384 | 8 | | 5 | out of memory | |
| YOLOv53 (r3) | Model size | The number of CSP for bottleneck | The number of convolution kernels | | The number of network parameters (×Million) |  | |
|  | s | 1, 3, 3 | 32, 64, 128, 256, 512 | | 7.2 | 90.20% | |
|  | m | 2, 6, 6 | 48, 96, 192, 384, 768 | | 21.2 | 91.40% | |
|  | l | 3, 9, 9 | 64, 128, 256, 512, 1024 | | 46.5 | 90.60% | |
|  | x | 4, 12, 12 | 80, 160, 320, 640, 1280 | | 86.7 | 92.10% | |

# Table S3: Hyper parameters used in YOLOv5

**Table S3** Description and hyper parameters of the tricks applied on the YOLOv5 network

| Tricks | Description | Hyper parameters | Values |
| --- | --- | --- | --- |
| Image Flip | Image flip up-down (probability) | flipud | 0.5 |
|  | Image flip left-right (probability) | fliplr | 0.5 |
| Mosaic | Image mosaic (probability) | mosaic | 1 |
| Mixup | Image mixup (probability) | mixup | 0 |
| Focal Loss | Focal loss gamma (EfficientDet default gamma=1.5) | fl_gamma | 0 |
| HSV Augmentation | Image HSV-Hue augmentation (fraction) | hsv_h | 0.015 |
|  | Image HSV-Saturation augmentation (fraction) | hsv_s | 0.35 |
|  | Image HSV-Value augmentation (fraction) | hsv_v | 0.2 |
| Other hyper parameters | Initial learning rate (SGD=1E-2, Adam=1E-3) | lr0 | 0.01 |
|  | Final OneCycleLR learning rate (lr0 * lrf) | lrf | 0.2 |
|  | SGD momentum/Adam beta1 | momentum | 0.937 |
|  | Optimizer weight decay 5e-4 | weight_decay | 0.0005 |
|  | Warmup epochs (fractions ok) | warmup_epochs | 3 |
|  | Warmup initial momentum | warmup_momentum | 0.8 |
|  | Warmup initial bias lr | warmup_bias_lr | 0.1 |
|  | Box loss gain | giou | 0.05 |
|  | Cls loss gain | cls | 0.5 |
|  | Cls BCELoss positive_weight | cls_pw | 1 |
|  | Obj loss gain (scale with pixels) | obj | 1 |
|  | Obj BCELoss positive_weight | obj_pw | 1 |
|  | IoU training threshold | iou_t | 0.2 |
|  | Anchor-multiple threshold | anchor_t | 4 |
|  | Image rotation (+/- deg) | degrees | 0 |
|  | Image translation (+/- fraction) | translate | 0.1 |
|  | Image scale (+/- gain) | scale | 0 |
|  | Image shear (+/- deg) | shear | 0 |
|  | Image perspective (+/- fraction), range 0-0.001 | perspective | 0 |

# Video S1: Access to the BALFilter Reader via the customized Web

The access to the BALFilter Reader via the customized Web was demonstrated in **Video S1**.

The customized Web is a user-friendly interface to promote the wide application of the BALFilter Reader. The collected large-field images could be easily imported to the BALFilter Reader via the Web, followed by the initiation of inspection (running of BALFilter Reader). The running progress of the BALFilter Reader can be finished in ~2 min for each case. When the BALFilter Reader running completes, the detection result will be returned to the Web and can be easily viewed. Every single image block can be easily addressed and viewed in the Web, which provides an easy access for the pathologists to view the adjacent cells/microenvironments around the predicted cells and thus better check the correctness of inference of the BALFilter Reader. Then, the double-checked objects (predicted tumor cells or background cells by the BALFilter Reader) can be submitted/added into the original dataset via the link provided in the Web, which is a simple and efficient way to further expand the dataset with more clinical samples in future.

To watch the video of operating the BALFilter Reader, please go as the following steps: 1) double click the following icon of Video S1; 2) watch the video in the popup window or save the video to a local disk and then watch.

# References

1. Pytorch Vision Resnet, https://www.pytorch.org, (accessed April 12, 2023).
2. Tan, Mingxing et al. “EfficientDet: Scalable and Efficient Object Detection.” *2020 IEEE/CVF Conference on Computer Vision and Pattern Recognition (CVPR)* (2019): 10778-10787.
3. Tips for Best Training Results - YOLOv8 Docs, https://docs.ultralytics.com/yolov5/tips_for_best_training_results/#dataset, (accessed April 10, 2023).
